# Identification of increased risk of perinatal anxiety: a multi-perspective qualitative study

**DOI:** 10.3399/BJGP.2024.0691

**Published:** 2026-04-08

**Authors:** Tamsin Fisher, Tom Kingstone, Noureen Shivji, Katrina Turner, Carolyn A Chew-Graham, Holly Christina Smith, Charlotte Archer, James Bailey, Jonathan Evans, David Kessler, Irene Petersen, Janine Proctor, Dahai Yu, Pensee Wu, Victoria Silverwood

**Affiliations:** 1 School of Medicine, Keele University, Keele, UK; 2 Midlands Partnership University NHS Foundation Trust, Stafford, UK; 3 Bristol Medical School, University of Bristol, Bristol, UK; 4 Research Department of Primary Care and Population Health, University College London, London, UK; 5 Department of Clinical Epidemiology, Aarhus University, Aarhus, Denmark; 6 Just Family CIC, Stoke on Trent, UK

**Keywords:** perinatal anxiety, qualitative research, risk, electronic health records, primary health care, general practice

## Abstract

**Background:**

Perinatal anxiety (PNA) is experienced by about 21% of women throughout the perinatal period. Identifying women at risk of PNA through primary care patient records could enable early intervention to improve treatment outcomes. The acceptability of doing this, however, is unknown.

**Aim:**

To explore patients’ and practitioners’ views on identifying women at risk of developing PNA using primary care patient records.

**Design and setting:**

Qualitative data are presented from a mixed-methods study, which used online and in-person interviews that were conducted in England.

**Method:**

Semi-structured interviews were held with 19 women with lived experience of PNA and 27 healthcare professionals (HCPs). Data were analysed thematically. A patient and public involvement and engagement group were involved throughout the study.

**Results:**

Both women and practitioners thought it was acceptable to identify women at increased risk of PNA using medical records, providing that sufficient acceptable help and support was in place. All participants also highlighted that an increased risk of PNA needed to be communicated sensitively, with women preferring phrasing such as ‘more vulnerable’ or ‘more susceptible’ than ‘high risk’. Challenges with identifying risk factors within patient records, such as limited sharing between HCPs and poor coding, were discussed by practitioners.

**Conclusion:**

There are challenges to identifying risk factors within patient records. It was felt that not all possible risk factors would be recorded in primary care records. The issues of limited sharing between HCPs and poor coding were discussed by practitioners, many of whom thought that clinical intuition was a more appropriate way to assess risk.

## How this fits in

Perinatal anxiety (PNA) affects approximately one in five women. Identifying women at risk of PNA could help them to engage with services earlier to better equip them to manage PNA. This study presents the qualitative findings, including perspectives of healthcare professionals (HCPs) and women with lived experience of PNA, from a mixed-methods study that examined the acceptability of using primary care records to identify women at risk of PNA. Women and HCPs felt it acceptable to use electronic health records to identify women at increased risk of PNA; however, identifying women at risk of PNA should not be completed without a plan for support to mitigate and/or limit the impacts of PNA.

## Introduction

Perinatal anxiety (PNA) is anxiety experienced during and up to 1 year after birth.^1^ The prevalence of PNA during the perinatal period is estimated to be ~21% globally.^2^ PNA has been associated with adverse pregnancy outcomes, such as miscarriage and pre-term delivery, challenges relating to mother–child bonding, and ongoing risk of maternal mental health problems.^3^ PNA has also been associated with postpartum depression.^4,5^

It has been estimated that between 8% and 30% of women needing psychological treatment for perinatal mental health problems are receiving support compared with those women receiving mental health support outside of the perinatal period (50%).^6^ This might be partly related to many women with PNA never receiving a formal diagnosis,^7^ or because practitioners may struggle to identify symptoms of women at risk of developing PNA. Early identification of women at risk of PNA could allow early intervention (for example, pharmaceutical, peer support, or support from community organisations), which could have health benefits for both parent and infant.

Currently, there is no validated recommended screening tool in the UK specific to PNA. The National Institute for Health and Care Excellence clinical guideline on antenatal and postnatal mental health recommends the use of the Generalised Anxiety Disorder 2-item (GAD-2) to screen for PNA at routine perinatal appointments,^8^ but some suggest it may not be sufficiently specific to PNA, resulting in lower identification of PNA.^9^

Electronic health records (EHRs) of patient data are widely used by GPs and other healthcare professionals (HCPs), such as advanced nurse practitioners and midwives, to identify risk for different health conditions such as cardiovascular disease (QRisk)^10^ and cancer.^11^ Risk models used in primary care have the potential to *‘influence clinical decision-making’*.^12^ When used in practice, HCPs need to have received appropriate training to use and interpret the results of such risk models. The risk models aim to predict the risk of future outcomes using available clinical parameters,^13^ such as demographics, diagnostic results, previous medical history, and prescribed medication history;^12^ however, challenges exist, including inconsistently coded data and lack of shared records.^14,15^ We explore some of these challenges in this study; the focus is not on the use of a prediction model owing to these challenges.

Early identification of PNA may lead to more positive outcomes for mother and baby. Therefore, it may be beneficial to develop a prediction model that uses EHRs to identify women at risk of PNA. However, before developing such a tool, to inform its use in practice, we need to know whether it would be acceptable to patients and practitioners.

This aim of this study was to explore patients’ and practitioners’ views on identifying women at risk of developing PNA using primary care patient records.

## Method

We are presenting qualitative findings from a mixed-methods study. The qualitative data collection adopted a post-positivist methodology. Semi-structured in-depth interviews were conducted with three groups: women with lived experience of PNA; HCPs working with women with lived experience of PNA; and Voluntary, Community and Social Enterprise (VCSE) representatives (VCSE data are not represented in this article).

All participants were purposively recruited via personal and professional networks, public mental health (PMH) networks, social media, and snowballing. Participants were invited to contact the research team to express interest in the study and subsequently received a participant information sheet. Recruitment was informed by patient and public involvement and engagement contributors (also referred to as the Patient Advisory Group; PAG) and the Clinical Advisory Group (CAG) who suggested individuals and organisations to contact to identify appropriate participants. Inclusion and exclusion criteria are listed in Box 1.

**Box 1.** Inclusion and exclusion criteria for interview participants

Inclusion criteria	Exclusion criteria
**Women with lived experience**	
Aged ≥18 years Living in the UK Lived experience of perinatal anxiety in the past 2 years from the time of the interview	No experience of perinatal anxiety Experience of perinatal anxiety >2 years from time of the interview, or before March 2020^a^
**Healthcare practitioners**	
Working in the UK Working with women with lived experience of perinatal anxiety	No experience of working with women with lived experience of perinatal anxiety

^a^Interviews began in 2022. We acknowledge that services and processes have changed since the start of the COVID-19 pandemic and wanted to reflect this in the data.

A topic guide was designed for each participant group (HCPs and women; see Supplementary Information S1). Topic guides included questions about interventions for PNA. These interviews were completed in collaboration with the senior author, who was collecting data on the same population for their PhD. Interviews were combined to include topics on intervention and high risk. This study is reporting on data relating to risk only, not interventions. Topic guides were developed in line with the aim of the interviews and informed by discussions with the PAG and CAG. These guides were revised according to iterative analysis of data, and prompts included in the topic guide are listed in Box 2.

Interviews were conducted via video call (Microsoft Teams) and in person depending on the participants’ preferences. They were conducted by the first author (research associate with a PhD in social geography), third author (research associate with a PhD in nursing), and the senior author (PhD Wellcome Trust Fellow and clinical GP), all of whom were experienced in qualitative interviewing. The research team have a multidisciplinary background (clinical and social sciences), which encouraged a more nuanced understanding of diverse perspectives.^16^ The interviews were audio-recorded, transcribed verbatim by an external company, and then anonymised by the first, third, and senior authors.

**Box 2.** Themes and topic guides of participant interviews

Participants	Topic guide prompts
Women with lived experience of perinatal anxiety	Lived experiences of perinatal anxiety Pre-disposing factors for perinatal anxiety Acceptability of using electronic health records to identify increased risk of perinatal anxiety Communication of risk (including who would be best suited to communicate an increased risk of perinatal anxiety)
Healthcare professionals working with women with lived experience of perinatal anxiety	Experiences of working with women with perinatal anxiety Pre-disposing factors for perinatal anxiety Acceptability of using electronic heath records to identify increased risk of perinatal anxiety Barriers to and facilitators of technology for accessing patient information Communication of risk

Each participant was given an identifier according to their role in the research; for example, women with lived experience are ‘W00n’ and HCPs are ‘HCP00n, job title’. Data were analysed thematically using a reflexive approach.^17^ Analysis informed ongoing interviews until no new themes arose from interviews and data saturation was met.^18,19^ Analysis was led by the first, third, and senior authors. Themes were discussed and agreed with all other co-authors, and members of the PAG and CAG.

Verbal and written consent were obtained before the interviews took place.

PAG members and the CAG contributed to multiple aspects of this research, from the funding application through to data analysis. Several team members have clinical experience of using medical records in primary care (fifth, ninth, tenth, and senior authors).

## Results

Interviews were held with 19 women (video call, *n* = 15; in person, *n* = 3; and telephone*, n* = 1) who had lived experience of PNA within the past 2 years and were aged between 23 and 41 (mean 30.7) years (Table 1). Although we did not set out to sample occupation, through the course of the study, some women disclosed that they were medical professionals (nurse, *n* = 3 and GP, *n* = 1). All of these women were interviewed as women with lived experience and their profession has little-to-no impact on the analysis; however, these women did have more accurate understanding of the challenges of EHRs and healthcare systems.

**Table 1.** Characteristics of women (*N* = 19)

Characteristic (as reported by participants)	*n*
**Age, years**	
20–25	2
26–30	5
31–35	8
36–40	3
41–45	1
**Ethnicity**	
Black	5
British Asian	1
Mixed race	1
Pakistani	2
White	5
White British	5
**Relationship status**	
Co-habiting	2
Engaged	2
Married	12
Single	3
**Children**	
Pregnant + 1 child	1
Pregnant + 2 children	2
1	10
2	5
3	0
4	1
**Geographical location**	
Bristol	1
Cambridgeshire	2
London	2
North Yorkshire	2
Staffordshire	10
Warwickshire	1
West Yorkshire	1
**Employment status**	
Employed (full time)	11
Employed (part time)	3
Not employed	2
Self-employed	1
Stay-at-home parent	2

Twenty-seven HCPs were interviewed (Table 2) and their years of clinical experience ranged from 6 months to 40 years. Only one HCP worked outside of the NHS as a private health visitor. All had worked with women with PNA in some capacity. All HCPs were practising across England. Twenty-five interviews were conducted by video call, and two interviews were conducted in person.

**Table 2.** Characteristics of healthcare professionals (*N* = 27)

Characteristic (as reported by participants)	*n*
**Job role**	
Advanced nurse practitioner	1
Community mental health nurse	2
GP	10
Health visitor	3
Healthcare worker	1
Junior doctor	1
Midwife	4
Perinatal mental health midwifery assistant	1
Perinatal mental health psychologist	2
Psychiatrist	1
School health nurse (and health visitor)	1
**Ethnicity**	
Black African British	1
Indian British	3
Irish British	1
Pakistani Canadian	1
White British	18
White non-British	1
White other	1
Did not disclose	1
**Geographical location**	
Birmingham	4
London	1
Manchester	3
Nottinghamshire	1
Oxfordshire	1
Staffordshire	15
Yorkshire	1
Did not disclose	1
**Age, years**	
20–29	1
30–39	12
40–49	8
50–59	5
60–69	1
**Gender**	
Woman	21
Man	6

Interviews for the women and HCPs lasted between 14 and 99 minutes (mean 57) and 38 and 76 (mean 43.3) minutes, respectively. Some HCP interviews were cut short owing to clinical commitments and time constraints.

The following three main themes were identified across the three datasets: identifying increased risk of PNA; communicating risk; and challenges of using EHR to identify women at risk. The last theme was divided into the following two sub-themes: system-level barriers; and individual-level barriers. Below, quotes have been used to illustrate some of the points made.

### Identifying increased risk of PNA

Women differed in their views about being identified as at high risk of developing PNA. Some women said that they were undaunted, explaining that they were used to hearing about different aspects of ‘risk*’* during pregnancy:

*‘Because they say that, in terms of, like, if there’s health conditions of, like, the mum, they say it’s a high-risk pregnancy, don’t they?’* (W009, aged 20–29 years)

Other participants recognised the importance of identifying women at risk, providing they had sufficient acceptable help and support in place, or could be directed to more support:

*‘The idea is not bad. It’s a nice idea … if it’s been done, it’s really going to help a lot of women out there that do not even have that support from family or friends.’* (W002, aged 30–39 years)

Most HCPs thought that identifying women at increased risk of PNA could be a good thing: several spoke about the benefits of early identification:

*‘I think that anything that helps that identification is going to be helpful … I think we’re doing a poor job of identification right now.’* (HCP04, psychologist)

HCPs suggested that women would appreciate being identified as high risk and said early identification was key to ensuring women received the most appropriate and timely care and management plans for PNA:

*‘I think it’s not just identifying women, but you could also use it to reduce that risk as well. You know, if they’ve got that kind of predisposing anxiety, how you can make that birth less traumatic if they’re already an anxious person. How can we alleviate those anxieties, how can we make sure this is not traumatic for you? Yeah, prevention as well as looking at interventions afterwards as well.’* (HCP16, mental health nurse)

While most people were accepting of being identified as having a higher risk of PNA, some women and HCPs expressed a variety of reservations and concerns. A couple of women thought that being told they were at higher risk of developing PNA could become a ‘trigger’ for then developing it. Some women reflected on the impact this might have, for example, on their ability to work:

*‘I don’t really want anxiety on my record because I don’t want it to affect my job even though it wouldn’t affect my job, but you’ve still got in your mindset that you really might have to be off sick with it and that kind of thing.’* (W016, aged 30–39 years)

In addition, GPs were cautious not to overburden themselves with an already high caseload, and other HCPs shared the concern that they might not be able to support women as their resources and time were limited, and there were limited referral options:

*‘I think that, you know, if somebody’s been able to disclose, there needs to be some skills on how to validate and make sure that the woman’s safe before she leaves. I think that would be my main concern. And of course that comes with time, the time that may not be available.’* (HCP013, specialist midwife)

Some GPs mentioned that they were familiar with using EHRs to identify risk scores, such as QRisk for cardiovascular disease, and therefore thought a risk score for PNA would be acceptable. However, they identified an ethical dilemma — if someone was identified as high risk, HCPs would be obliged to communicate this risk to the patient and act on it:

*‘Obviously, you have to be very sensitive around any mental health problems. You don’t know what the person has been through previously and it could be triggering. I think once a patient has been identified, it would be vital to notify the patient ... what algorithm has said so* [audio cut out] *me being supportive.’* (HCP01, GP)

### Communicating risk

Multiple participants, including women and HCPs, commented that if women were to be identified as ‘high risk’ for PNA, it would be important to consider the way in which risk was communicated. Some women felt the term ‘high risk’ was quite frightening and that alternative language could be considered, such as, ‘more vulnerable’, ‘more likely’, or ‘more susceptible’:

*‘High risk is not appropriate I think. The word is susceptible. “You might develop anxiety if you don’t care about yourself, this will end up in this scenario.*” *I think the correct word is prone, susceptible, vulnerable. I think vulnerable is a good word.’* (W018, aged 20–29 years)

However, some of the women felt that avoiding the term ‘high risk*’* was not necessary, as it was a word commonly used during pregnancy in relation to other tests they underwent (for example, anomaly scans and risk of pre-eclampsia). One woman commented that ‘vulnerable’ may insinuate the woman was not capable:

*‘Vulnerable to me would mean that I’m really out of control, I can’t manage.’* (W016, aged 30–39 years)

Women shared a variety of views about which HCP would be most appropriate to communicate risk of PNA. They explained what was important was the patient’s relationship with different HCPs and their level of trust in them. Some women suggested that midwives might be best suited to communicating risk of PNA, as they had regular contact and continuity with women. Others thought that their GP might be more appropriate:

*‘I would say GP, because they are the one that is still there when you have the baby.’* (W016, aged 30–39 years)

GPs acknowledged that they had limited scheduled, routine opportunities to support perinatal women, as the only regular contact they had with perinatal women was the maternal postnatal consultation. One GP stated that while they didn’t have capacity, it was *‘a massive loss for GPs not doing antenatal care’* (HCP10, GP). GPs also felt they were not always best placed to communicate the risk of PNA owing to infrequent contact with women during the perinatal period compared with midwives and health visitors:

*‘I think that’s the reason I said midwife, health visitors was just because they have the most contact with these ladies … But then the GPs are well placed in the sense that some of them already know the patients, so they would already have that kind of information anyway. So, I think that it’s good if both can, but I also am aware that if you leave it to the GPs, it will fall through, probably because of the time pressures more than anything else.’* (HCP22, GP)

An ANP working for a PMH service suggested they did not feel GPs would have the capacity to perform such an assessment on top of their current workloads and, for this reason, thought that, along with midwives and health visitors, ANPs could communicate an increased risk of PNA.

### Challenges of using EHRs to identify women at risk

Interviewees’ accounts highlighted potential challenges of using EHRs to inform current practice, and these related either to the system or the individual (practitioner and women or patient).

#### System-level barriers

Practitioners explained that access to patient information was dependent on where care was being delivered and access to data systems. For example, community midwives who were based within a general practice may have access to their patients’ primary care EHRs. However, hospital midwives were unlikely to have access to primary care records but would have access to records shared by community midwives and some other health professionals, such as health visitors, from within their trust. As such, there would be no guarantee that all midwives and health visitors would have access to the same data and/or be aware that a woman was at increased risk of developing PNA:

*‘Most likely, however it depends what computer systems we’re using. For example, if I was doing a consultation in a GP surgery and I’d got access to the GP records, then I could perhaps see on those GP records that she’s been medicated on, you know, antidepressants. Whereas if she comes to me at the* [hospital] *for example … If she came to a hospital setting, then that midwife may not have access to that information and that could again go missing.’* (HCP05, community midwife)

Hospital midwives advised that they did not have access to GP records and therefore were not able to view a woman’s full history that may include predisposing risk factors for PNA.

HCPs described how the note systems used by different HCPs did not always link together and, therefore, notes were spread across multiple platforms. Furthermore, GPs noted that they did not always have access to their patients’ birth records. For example, a traumatic caesarean section would not be coded in EHRs by hospital midwives, although the information might, in some cases, be accessible as written notes:

*‘Previous traumatic birth, I don’t know how reliably previous traumatic birth is coded in a primary care record, I suspect not very, not very by me … We’ve kind of done it in the postnatal, definitely in the six-week check and, you know probably write it in free text and things like that. But probably won’t code it.’* (HCP11, GP)

Instead of relying on primary care records, some HCPs suggested they would prefer to use their clinical intuition when working with women they thought may be at increased risk of PNA:

*‘I think the midwife has to also exercise her clinical expertise.’* (HCP06, community midwife)

#### Individual-level barriers

Two women and one HCP commented that women should consent to their EHRs being used to identify potential risk of PNA, as not securing their consent would be disrespectful and an invasion of privacy:

*‘I think it’s wonderful, it’s actually a great, great idea, it would probably would help a lot of people. But I want to know would this be done with the permission of the patient or without permission of the patient? ... I think you should be able to ask patients before doing it, because contrary to the fact I’m a private person, I would find that very annoying.’* (W003, aged 30–39 years)

One HCP stated they were not comfortable looking through records to identify predisposing factors for PNA and would therefore prefer to use clinical judgement:

*‘I’d be more tempted to use my clinical expertise and knowledge that I’ve learned through training than trawl somebody’s records because I don’t feel that that’s right.’* (HCP05, community midwife)

Not all women felt that consent was essential, and some were *‘indifferent’* (W004) about their records being used to identify increased risk of PNA. Finally, some women may fear disclosing anxiety or depression and therefore it would not be recorded in their records:

*‘I think it’s always worth, you know, kind of identifying out someone’s record. And you know, I don't know how you would broach discussing it, but just …. someone may not feel comfortable about disclosing their fears and or, you know, their concerns about, you know, anxiety or, you know, depression.’* (W014, aged 30–39 years)

## Discussion

### Summary

Our research suggests that HCPs felt that EHRs could be used to identify women at higher risk of PNA. They suggested, however, that clinical intuition was also important, and there were limitations with EHRs. Some women were concerned that being identified as ‘higher risk of PNA’ may cause them to become more anxious. Other participants suggested that identifying women ‘at risk*’* of PNA could in fact help prevent PNA and/or provide an opportunity to work with patients to develop a plan to prevent or manage the impacts of PNA.^20^ Identification and communication of risk should be followed by an offer of appropriate support.

There was no consensus on who should communicate risk, although secondary care services, such as perinatal mental health nurses, suggested they would be well placed to support women at risk of PNA and to relieve some pressures of GPs. GPs have limited contact with most women during pregnancy and in the first few days and weeks after childbirth; women are directed to self-refer into community midwifery teams at the antenatal stage, and the only planned care they have with their GP is at the 6-week postnatal check.

Finally, HCPs identified the limitations to using EHRs including inconsistencies and inaccuracies in the way in which data are coded. While most women felt it may be useful to be identified as higher risk, some women admitted that their PNA was not recorded on their records and/or they withheld information. Some women and HCPs felt it might be acceptable to identify people at increased risk, but that patients should be asked before using EHRs to identify said risk in the same way that people must, for example, consent to testing.

### Strengths and limitations

Interviews were held with women who varied in age, ethnicity, number of children, relationship status, and location in the UK. The ethnic diversity of the women was a real strength given the current ethnic disparities in mental health care and perinatal health care.^21^ The HCPs interviewed differed in terms of their professional backgrounds, age, and experience. However, there was a lack of ethnic diversity (most HCPs were White British), geographical location in the UK (specifically from the south of the UK), and gender among HCPs. Some HCP interviews were cut short owing to clinical commitments and constraints on time.

We did not collect information about occupations of women; however, it is important to highlight that one of the women had both clinical and lived experiences of PNA. Three of the women had clinical experience; however, this was unrelated to PNA.

This was a mixed-methods study, and by using semi-structured interviews we were able to identify potential challenges of using EHRs that were not identified in the quantitative component of this study. Challenges included the difficulties in recording patient information given EHRs are becoming increasingly accessible to patients; identifying the challenges and ethical implications of the use of risk predictor models; and highlighting the voice of the patient (women with lived experience of PNA) and their opinions and apprehensions of the use of their EHRs.

### Comparison with existing literature

Health care today is increasingly reliant on new information technology systems to store and transfer patient information to increase and improve decision making.^22^ The lack of integration of electronic health systems within and between HCPs and trusts is understood to be a barrier to identifying and treating multiple health conditions.^12^ The data presented in this study demonstrate that integration, or lack of, may also pose a challenge when considering the possibility of identifying women at increased risk of PNA. Some women do not disclose anxieties ante- and postnatally for fear of repercussions; for example, at work (EHRs are not accessible to employers without permission from the patient [employee]). HCPs noted that they sometimes have limited access to patients’ EHRs and primary care data, which could prevent them from supporting women as necessary during appointments for women or infants.

Previous research into the acceptability of using EHRs to identify mental ill-health are quantitative in nature;^23^ however, the literature on the acceptability of using EHRs to identify mental ill-health is limited.^23^ This study, therefore, contributes to better understanding the acceptability of using EHRs to identify risk of PNA. Women discussed the use of alternative language and appropriate methods of communicating the risk. Some suggested language such as *‘more susceptible to’* or *‘more vulnerable to’* was more acceptable than *‘high risk’*. There is a wealth of literature addressing how risk is communicated to patients;^24,25^ however, there is very little to address the patient’s preference, especially the language and terminology used. Much like previous research, women valued communication of risk with HCPs that they knew and had good rapport with.^26,27^ Literature exists around the communication of risk via images;^25^ however, little research looks at the language of communicating risk.

### Implications for research and practice

Our study suggests that women and HCPs feel that it would be acceptable to use EHRs to identify women at increased risk of PNA. There are systemic and individual-level barriers to patient information and data available within these records. HCPs highlighted the challenges of inconsistent data systems across the NHS, while women with PNA do not always disclose their struggles with their GP. The use of a risk prediction model for PNA will require quantitative analysis to develop the model, and further research could be performed to further understand patients’ perspectives of the use of such models. Finally, identifying women at high risk of PNA should not be completed without a plan for support to mitigate and/or limit the impacts of PNA.
